# Movement Kinematics and Interjoint Coordination Are Influenced by Target Location and Arm in 6-Year-Old Children

**DOI:** 10.3389/fnhum.2020.554378

**Published:** 2020-09-16

**Authors:** Leia B. Bagesteiro, Rogerio B. Balthazar, Charmayne M. L. Hughes

**Affiliations:** ^1^Department of Kinesiology, San Francisco State University, San Francisco, CA, United States; ^2^Pós-Graduação em Neurociência e Cognição, Universidade Federal do ABC, Santo Andre, Brazil; ^3^Health Equity Institute NeuroTech Lab, San Francisco State University, San Francisco, CA, United States

**Keywords:** development, kinematics, interjoint coordination, manual asymmetries, children

## Abstract

Rapid aiming movements are typically used to study upper limb motor control and development. Despite the large corpus of work in this area, few studies have examined kinematic manual asymmetries in children who have just started formal schooling and until now, none have characterized how children coordinate their joints to complete these movements (i.e., interjoint coordination). In the present study, manual asymmetries in kinematics and interjoint coordination in strongly right-handed 6-year-old children were investigated when reaching for ipsilateral and contralateral targets with their dominant right arm and the non-dominant left arm. Overall, manual asymmetries in interjoint coordination are apparent for both 6-year-old children and young adults, although young children completed the task by adopting a different strategy than adults. Also, control strategies employed by 6-year-old children were influenced by both the location of the target as well as the arm used to perform the task. Specifically, compared to all other conditions, children’s trajectories were more curved when performing contralateral movements with the non-dominant left arm, which were driven by smaller shoulder excursions combined with larger elbow excursions for this condition. Based on these results, we argue that the differences in interjoint coordination reflect the stage of development of 6-year-old children, the origin of which derives from maturational (e.g., hand dominance) and environmental factors (e.g., school-based experience).

## Introduction

The manner in which the neuromotor system plans and controls goal-directed aiming movements with the dominant and non-dominant arm has been an interest of researchers for more than 125 years. In the now seminal studies of Woodworth (1899), it was found that repetitive line drawing movements performed by the dominant right arm were substantially more accurate than those of the non-dominant left arm and that the degree of manual asymmetry became more pronounced at faster movement speeds. Since Woodworth, research from various experimental paradigms has revealed that the dominant right arm of right-handed adult individuals is faster to place pegs into target holes during tasks with high precision demands (Woodworth, 1899; Annett et al., 1979; Todor and Kyprie, 1980; Boulinguez et al., 2001), exhibits less movement variability (Peters, 1976; Todor and Kyprie, 1980; Elliott et al., 1986), and generates more force than the non-dominant left arm (Provins, 1967; Brouwer et al., 2001; Farthing et al., 2005).

Similarly, research in typically developing populations has also revealed a dominant hand advantage in discrete unimanual reaching (Schneiberg et al., 2002), manual dexterity (e.g., peg placing (Annett, 1970), threading nuts on bolt (Pedersen et al., 2003), finger-tapping (Carlier et al., 1993), and drawing tasks (van Mier, 2006). For example, Annett (1970) used spatiotemporal techniques to evaluate manual asymmetries in children between 3- to 15-years old when performing a peg placing task. Results indicated that the preferred right arm performed the task faster than the non-preferred left arm (irrespective of age) and that the degree of manual asymmetry remained constant across development. In a more recent study, van Mier (2006) employed kinematic analysis to examine movement asymmetries in discrete and continuous drawing tasks in children between 4 and 12 years of age. Results demonstrated that children were able to perform a discrete movement task more accurately and efficiently (i.e., drawing distances) when they used their dominant, compared to the non-dominant arm. However, the percentage of stop time (i.e., percentage of task with zero velocity) was smaller when performing the task with the non-dominant hand, which the authors hypothesize is due to improved proprioception to the non-dominant hand control system. They then point to research by Sainburg and Kalakanis (2000)—one of the studies that contributed to the dynamic dominance hypothesis (Sainburg, 2002)—in which hand trajectories and joint coordination patterns support the idea that the dominant arm is proficient for muscle and interjoint interaction, while the non-dominant arm is more adept at using proprioceptive feedback.

Interestingly, there is growing research indicating that the difference in performance between the hands is influenced by the degree of precision and skill required to complete the task (Annett, 1992; Bryden and Roy, 1999; Schulze et al., 2002; Bryden et al., 2007). For example, in Bryden et al. (2007), individuals performed a peg placing task, in which the difficulty of the task was manipulated by adjusting the precision requirements when picking up the peg, inserting the peg, or both. Results indicated that the task was completed more quickly when the dominant right arm was used and that this difference was more pronounced when the final precision demands were high compared to when precision demands are low.

In sum, studies on manual asymmetries in children have focused on spatiotemporal kinematic performance (e.g., movement time, peak velocity, accuracy) between the two arms. In contrast, there has been little work that has characterized how children coordinate their joints to complete discrete movements (i.e., interjoint coordination, but see Schneiberg et al., 2002), and distinctly less that has described differences in interjoint coordination between the two limbs. This is unfortunate given that stabilization of the end-effector trajectory necessitates adequate coordination between the shoulder and elbow to meet task goals (Morasso, 1981), and that the trajectory of the hand is critically dependent on interjoint coordination and control of intersegmental dynamics (Sainburg et al., 1993, 1999; Ghez and Sainburg, 1995).

In one of the only studies to examine interjoint coordination in normally developing populations (Schneiberg et al., 2002), children between 4 and 11 years of age performed reaching movements to targets at three different distances (66%, 100%, and 166% of children’s arm length) with the dominant arm. Results of that study indicated that movement kinematics reached adult levels by 8–9 years of age, but that the temporal coupling of the shoulder and elbow (an indication of inter-joint coordination) did not reach levels typically observed in adult populations, regardless of age. Based on these results, Schneiberg et al. (2002) suggested that the development of skilled upper limb motor actions requires that children learn how to minimize the excessive degrees of freedom in the upper limb as well as find an interjoint coordination pattern that is most appropriate for the task. However, whether there exist manual asymmetries in interjoint coordination has not been fully investigated in developing children.

In addition to effects due to arm, there is a wealth of literature demonstrating that target location has a significant effect on reaching kinematics and interlimb coordination, with the bulk of research indicating that movements to targets located on the same side of the body as the reaching limb (ipsilateral) exhibit greater endpoint accuracies, shorter movement time, higher peak velocities when compared with movements to targets located on the opposite side of the body midline (contralateral; Carson et al., 1993; Elliott et al., 1993; Carey et al., 1996; Hodges et al., 1997). Ipsilateral target advantages have also been reported in developing children (Smits-Engelsman et al., 2004; Zoia et al., 2005). For example, Smits-Engelsman et al. (2004) had 48 right-handed children between 6 and 10 years of age perform rapid aiming movements toward targets positioned either at the midline, contralateral or ipsilateral hemispace. Findings revealed that movements were more accurate in ipsilateral than in contralateral space and that older children were more accurate, faster, and made smoother aiming movements than younger children.

Interestingly, there is evidence that the ipsilateral advantage is present in full-term infants as young as 6 months of age (Morange-Majoux et al., 2000; Hopkins and Rönnqvist, 2002; Rönnqvist and Domellöf, 2006). Using a longitudinal study, Rönnqvist and Domellöf (2006) examined right, left, or midline position reaching in infants over the ages 6, 9, 12, and 36 months. Results indicated that full-term infants consistently exhibited straighter and less segmented trajectories when making right-sided reaching movements, which improved with increasing age. Based on these results, the authors argue that the presence of kinematic differences when reaching to targets in ipsilateral and contralateral hemispace are present long before infants have developed movement patterns based on experience, and thus reflect biologically based developmental processes.

Motivated by this work, the present study aimed to characterize control strategies (i.e., how the neuromotor system controls the sequences of movements organized to accommodate a behavioral problem that requires many steps in its solution—Profeta and Turvey, 2018) employed by strongly right-handed 6-year-old children when executing a unimanual planar rapid aiming task, and to determine whether there exist manual asymmetries in kinematics and interjoint coordination. Besides, we also compare the data of 6-year-old children to that of healthy young adults (as reaching abilities of this population is indicative of upper bound performance) to determine which reaching metrics are fully matured at this stage of development.

Based upon the wealth of literature that has examined manual asymmetries in upper limb *kinematics* from both developing and adult populations, we expect that movements performed by the dominant right arm will be faster and more accurate than movements performed by the non-dominant left arm, irrespective of group. Moreover, for both adults and children, we expect that movements will be faster, and performed with greater elbow excursion (but smaller shoulder excursion) values for the ipsilateral target than the contralateral target.

Concerning kinematic differences based on the target location, we hypothesize that movements to the ipsilateral target will be faster and feature mainly elbow excursions compared to the contralateral target, for both adults and children. In contrast, movements to the contralateral target will present large intersegmental effects (i.e., using both shoulder and elbow similarly), with more curved hand paths for the non-dominant left, compared to the dominant right arm.

## Materials and Methods

### Participants

Forty-five healthy right-handed children (mean age = 77.6 months ± 5.3, 19 male and 26 female) from two elementary public schools and 16 adults (mean age = 25.3 years ± 5.3, 10 male and six female) participated in the current study. Due to the purpose of the current study, pre-screening of potential participants was conducted *via* an online survey to determine their (or their children’s) initial eligibility for the study. Individuals were excluded from participation if they performed less than nine tasks in the handedness battery with their right hand, had any known neuromuscular disorders, and did not have normal or corrected to normal vision (as measured by the Snellen E-chart). Also, children were excluded if they scored below the 15th percentile on the Movement Assessment Battery for Children (M-ABC) age-class 1 (ages 4–6; Henderson and Sugden, 1992).

Before testing, handedness was determined using a 10-task questionnaire (e.g., drawing, throwing a ball, cutting with scissors, using a pencil sharpener, opening a box, et cetera). In addition to having the parents of the children complete the survey, we confirmed the manual preference information provided by the parents by asking all children to physically perform the tasks. All adult participants completed the Dutch Handedness Questionnaire (van Strien, 2003). Before participation, written informed consent (and assent in the case of children) was obtained from all participants. The experiment was approved by the Institutional Review Board at the *Universidade Federal do ABC* and San Francisco State University and was conducted following the declaration of Helsinki.

### General Experimental Paradigm

Unraveling the neuromotor control processes involved in multijoint movements (e.g., arm reaching) has been successfully examined with the use of target(s)-pointing paradigm. In these tasks, individuals perform aiming movements to targets located in ipsilateral and contralateral space as quickly and accurately as possible. Most often the task requires planar movements (see Sainburg and Kalakanis, 2000; Sainburg, 2002, 2014), but the paradigm has also been used to examine reaching in three-dimensional space (see Butler et al., 2010; Hung et al., 2012). Regardless of the exact setup, this paradigm allows researchers to record behavioral data regarding movement kinematics and muscle torques, as well as to research perturbation effects (Campolo et al., 2014).

The robustness of the target(s)-pointing paradigm is demonstrated by the wealth of studies that have used it to analyze differences in right and left arm performance (i.e., interlimb coordination, Sainburg and Kalakanis, 2000; Sainburg, 2002, 2014), motor learning (Singh and Scott, 2003; Kim et al., 2009; Crevecoeur et al., 2019) and motor adaptation (Salomoni et al., 2019; Coltman and Gribble, 2020) strategies in healthy young adults, and is bolstered by its use in a variety of different populations, including normal aging (Ketcham et al., 2004; Lee et al., 2007; Przybyla et al., 2011), stroke (Schaefer et al., 2009; Mutha et al., 2010; Laczko et al., 2017), Parkinson’s disease (Fradet et al., 2009), and developing children (Konczak and Dichgans, 1997; Lee et al., 2008).

### Experimental Setup and Procedure

**Figure 1:**
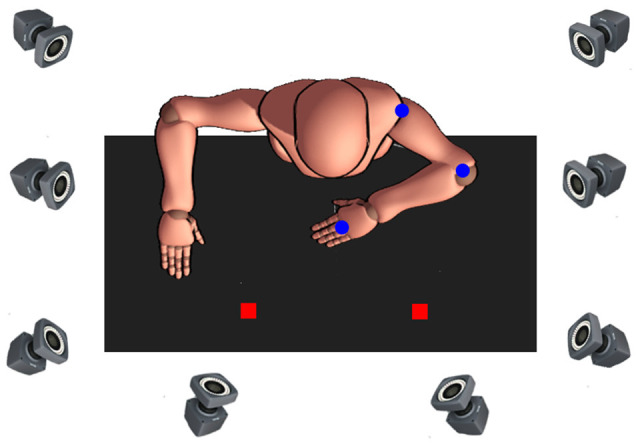
Bird’s eye view of the experimental setup. Depicted is the start position when movements were to be made with the non-dominant left arm. Passive markers (blue dots). Targets (red squares).

The experimental set-up was positioned on a height-adjustable table, upon four paper locations (2 × 2 cm) were taped flat to the surface and served to indicate the start and three target locations (see Figure 1). The start location was 10 cm from the edge of the table and vertically arranged to coincide with the participants’ body midline. Targets were oriented 45°, 90°, and 135° from the horizontal axis. This target arrangement allows a unique movement of each joint, for example, for movements performed with the right arm, 45° (ipsilateral) target requires almost exclusively elbow excursion, whereas 90° and 135° (contralateral) target movements require a distinctive combination of shoulder and elbow excursions. Also, the middle (90°) target was used to reduce expectancy effects and improve trial randomization throughout the experiment, which is especially important for the children, increasing motivation and task-attention (as revealed by our pilot testing). Due to differences in average arm length between children and adults (Fryar et al., 2016), the linear distance from each of the three targets was normalized, such that the distance from the start location to each target was 19 cm for children and 27 cm for adults. The manipulated object was a circular plastic puck (5 cm in diameter, 0.5 cm in height) that had a central pin (0.8 cm diameter, 4 cm in height). The base of the object was covered in felt cloth to reduce friction between the object and the table.

Reaching movements were recorded using an eight-camera optical motion capture system (Bonita 10, VICON Motion Systems; with a temporal and spatial resolution of 200 Hz and 1 mm, respectively) sampling at 120 Hz. Passive markers were then attached to the acromion process, lateral epicondyle of the humerus and head of the third metacarpal to create the biomechanical model of the arm consisting of two segments (lower arm and upper arm) and two joints (elbow and shoulder).

At the start of the experimental session, participants were seated in front of the height-adjustable ergonomic table with the arm of the reaching limb positioned at 90° to the trunk in the sagittal plane. To ensure that the non-reaching arm did not interfere with task performance, the shoulder and elbow of the non-reaching limb were positioned 180° and 90° relative to the trunk, respectively (see Figure 1). The wrist of the reaching arm was then immobilized using an adjustable orthosis to minimize radial and ulnar deviation, and the central pin of the puck was tightly placed between the 3rd and 4th fingers. The arm that was not performing the task rested at a 90° angle on the tabletop. Participants were informed that the task involved rapid aim movements and that they were to move the puck so that the marker on the 3rd metacarpal is as close to the center of the target as possible. Participants were instructed to “move as fast as you can, and if you pass the target, do not make any corrective movements.” At the start of each trial, the experimenter gave the verbal command regarding which target to aim for, after which the participants made a single, uncorrected, rapid aiming movement from the start position to the target. The participant remained at the final position for 2 s, then moved the puck back to the start location at a comfortable pace. We emphasized the speed of responding and the requirements that participants did not make any corrective movements or move their trunk or their head while reaching.

Each experimental block (dominant arm, non-dominant arm) began with a series of 25 trials (10 to the ipsilateral and contralateral targets, five trials to the center target) to familiarize the participant with the general task procedures and provide feedback about movement accuracy. Following the acclimatization trials, participants took a 2-min rest break and then performed 50 experimental trials. The factor arm was blocked, and half of the participants performed the task with the left arm first, while the other half performed the task with the right arm first. Within each arm block, participants performed 20 trials to the ipsilateral and contralateral targets, and 10 trials to the center (90°) target. The order of the target was fully randomized. The experiment took approximately 30 min to complete.

### Data Processing and Analysis

The 3D coordinates of the reflective markers were reconstructed and interpolated using a Woltring filter (5 mm^2^ predicted mean square error). Kinematic variables were calculated using custom MATLAB scripts (The MathWorks^®^, Version R2018). For each trial, only the period between when the hand first moved (movement onset) to the time the hand reached the final position (movement offset) was further analyzed. Movement onset was determined as the time of the sample in which the resultant tangential velocity (*x*- and *y*-axes) of the hand marker exceeded 3% of peak velocity, whereas movement offset was determined as the time of the sample in which the resultant velocity dropped and stayed below 3% of peak hand velocity.

Previous studies in healthy young adults (Sainburg and Kalakanis, 2000; Bagesteiro and Sainburg, 2002; Sainburg and Schaefer, 2004) have determined differences in right and left arm performance (i.e., patterns of multijoint/interlimb coordination), which is associated with changes in asymmetries in the trajectories and accuracies of reaching movements, as well as hand trajectory formation that underlies patterns of proximal joint motion (i.e., the shoulder and elbow). To examine how 6-year-old children coordinated multidirectional reaching movements in the horizontal plane they performed movements with different joint excursion requirements. Thus, our targets required an increasing amount of shoulder excursion while maintaining a similar elbow extension. Because of the projecting intersegmental dynamics of these motions, we focused our investigation on testing interlimb differences in hand path direction and curvature (i.e., deviation from linearity and ratio of shoulder and elbow excursions) as well as kinematic measures standardly reported in the literature (Kim et al., 2009; Laczko et al., 2017; Crevecoeur et al., 2019).

The five kinematics measures of planar aiming movements were calculated from the hand path of each trial: movement duration, final position error, peak hand tangential velocity, and elbow and shoulder excursion. Movement duration was calculated as the elapsed time from movement start to movement end. The final position error was calculated as the difference between the final position of the hand and the center of the aimed target. Peak hand velocity was the maximum value obtained from the hand position differentiation curve. Elbow and shoulder excursion were calculated as the difference between the final angular position and initial angular position from the elbow and shoulder angular displacement profiles, respectively.

Also, two interjoint coordination measures were calculated: deviation from linearity, and the ratio of shoulder and elbow excursions. Deviation from linearity was calculated based on hand path (i.e., hand marker trajectory) as the minor axis divided by the major axis of the hand path trajectory. The major axis was defined as the largest distance between any two points in the path, whereas minor axis was defined as the largest distance, perpendicular to the major axis, between any two points in the path (Bagesteiro and Sainburg, 2002). This measure quantifies the degree to which the hand path is linear (=0) or curved (>0), and considers the hand’s trajectory over the entire course of a movement. The shoulder/elbow excursion ratio was calculated by dividing shoulder excursion values by elbow excursion values, with values greater than one indicating that shoulder excursions were greater than elbow excursions, and values lower than one indicating that elbow excursions were greater than shoulder excursions.

### Statistical Analysis

After the 3D coordinates were reconstructed and tangential velocity profiles of the hand were calculated, we excluded trials performed in a non-instructed manner (moving before the start of the trial, moving to the wrong target), movements greater than 1,500 ms, or trials in which movement onset, peak velocity, and movement termination were not correctly determined. Error trials comprised less than 3% of the data and were approximately equally distributed across conditions and participants. Given the low error rate, mean substitution was used to replace missing values.

Statistical quantification of the differences in kinematic characteristics was conducted on five linear measures (final position error, movement time, peak hand velocity, shoulder excursion, elbow excursion) and two interjoint coordination angular measures (deviation from linearity, shoulder/elbow excursion ratio). For each dependent variable, the average of each condition was submitted to a Repeated Measures Analysis of Variance with Group (Children, Adult) as the between-subjects factor, and Direction (Contralateral, Ipsilateral) and Arm (Dominant, Non-dominant) as the within-subjects factor. Preliminary analyses were conducted to check for normality, sphericity (Mauchly test), univariate, and multivariate outliers. All data met the criteria for normality (*P* > 0.05) except for the shoulder/elbow excursion ratio. A log transform was used on these data and the criteria for normality were met; the transformed data were used for all statistical analyses. Data were collapsed across gender, as preliminary data analysis did not reveal any systematic differences between males and females (Flatters et al., 2014). Results with *p*-values < 0.05 were considered significant. Partial eta-squared ($\eta_{\text{p}}^{2}$) values were calculated for all *F-tests* as an indicator of effect size. Significant main effects and interactions were compared using Bonferroni corrected *post hoc* analysis.

## Results

Given that the primary aim of the study was to investigate possible interlimb (dominant right arm vs. non-dominant left arm) differences when performing aiming movements to two target locations that required differing biomechanical configurations (i.e., ipsilateral and contralateral targets), the results section is focused on interaction effects directly related to manual asymmetries, target location, and age. More detailed statistical reporting is provided in Supplementary Appendix Tables A1–A7). Table 1 summarizes (Means and SE) for our variables of interest for each group, arm, and target.

**Table 1:** Means and SE (in brackets) for variables of interest for each group, arm, and target.

	Ipsilateral target				Contralateral target			
	Dominant		Non-dominant		Dominant		Non-dominant	
	Adult	Child	Adult	Child	Adult	Child	Adult	Child
Movement duration (ms)	290 (130)	535 (80)	294 (150)	541 (90)	354 (160)	632 (100)	379 (190)	681 (110)
Final position error (cm)	1.97 (0.21)	2.00 (0.12)	2.60 (0.27)	2.05 (0.15)	2.45 (0.17)	1.82 (0.09)	2.05 (0.24)	2.31 (0.14)
Peak hand velocity (cm/s)	19.52 (0.67)	7.63 (0.40)	19.36 (0.68)	7.89 (0.04)	16.04 (0.50)	6.77 (0.03)	14.89 (0.50)	6.40 (0.30)
Shoulder excursion (°)	6.32 (0.83)	8.14 (0.49)	8.20 (1.03)	8.97 (0.62)	49.91 (1.42)	50.39 (0.85)	49.04 (1.24)	49.00 (0.74)
Elbow excursion (°)	41.59 (0.97)	51.78 (0.58)	37.07 (1.18)	53.61 (0.70)	63.20 (1.34)	15.53 (0.80)	64.62 (1.49)	17.90 (0.89)
Deviation from linearity	0.08 (0.005)	0.10 (0.003)	0.07 (0.006)	0.10 (0.004)	0.04 (0.004)	0.06 (0.002)	0.04 (0.005)	0.08 (0.003)
Log (shoulder/elbow	−0.87 (0.06)	−0.85 (0.03)	−0.65 (0.06)	−0.84 (0.4)	−0.01 (0.05)	0.54 (0.03)	−0.03 (0.04)	0.46 (0.3)
excursion ratio)								

### Movement Kinematics

**Figure 2:**
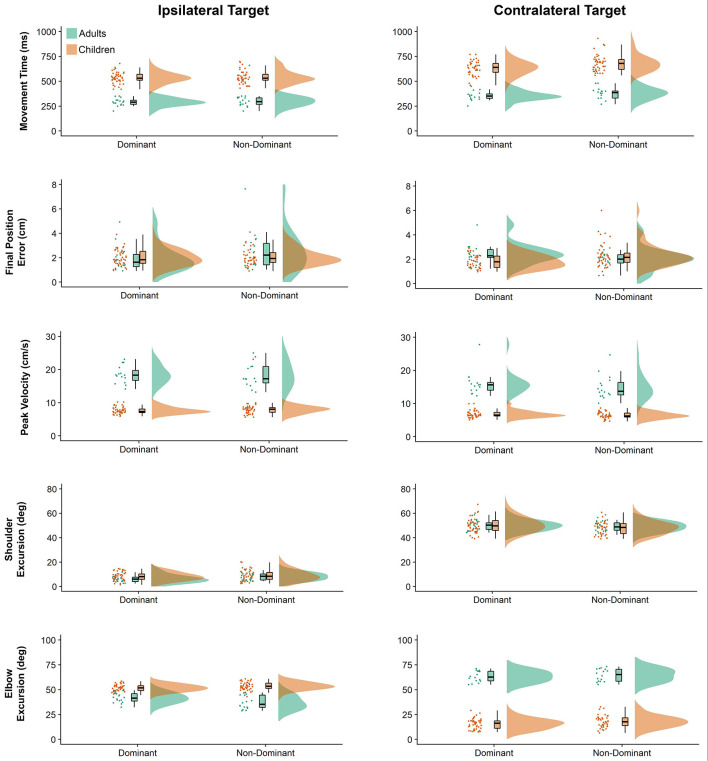
Raincloud plots showing the raw data, data distribution, and five summary statistics for movements to the ipsilateral (left panels) and contralateral directions (right panels) for linear kinematic variables. Data from preschool children is shown in brown, while data from young adults is plotted in green.

Raincloud plots (Allen et al., 2019) depicting raw data, data distribution, and five summary statistics (i.e., median, first quartile, third quartile, min, and max) for kinematic variables are presented in Figure 2. We found significant differences in movement time (*P* = 0.010) and final position error (*P* = 0.016) between the arms, indicating that the dominant arm took less time and was more accurate than the non-dominant arm. Also, movements performed to the contralateral target presented longer duration (*P* < 0.001), were slower (lower peak velocity, *P* < 0.001) and were completed with greater shoulder excursion (*P* < 0.001) and less elbow excursion (*P* < 0.001) as compared to the ones performed to the ipsilateral target. There was a significant difference in movement time (*P* < 0.001), peak velocity (*P* < 0.001), and elbow excursion (*P* < 0.001) between groups, showing that children took longer to move, reaching lower peak velocities and presented less elbow excursion as compared to adults. Children and adults exhibited longer average movement times (see Supplementary Table A-1) for the contralateral target than ipsilateral the direction, however, this difference was more pronounced for the children (mean difference = 118 ms) compared to the adults (mean difference = 74 ms). Although average movement time values were similar for the contralateral target regardless of the arm (dominant = 559 ms, non-dominant = 601 ms), movements to the ipsilateral target were much longer when performed with the non-dominant arm (dominant = 476 ms, non-dominant = 471 ms).

Children’s average final position error values (see Supplementary Table A-2) were similar for both arms when moving to the ipsilateral target (dominant = 2.00 cm, non-dominant = 2.05 cm), but larger for the non-dominant arm when moving to the contralateral target (dominant = 1.82 cm, non-dominant = 2.31 cm). In contrast, average final position error values for adults were larger for the non-dominant arm when moving towards the ipsilateral target (dominant = 1.87 cm, non-dominant = 2.64 cm), but larger for the dominant arm when moving towards the contralateral target (dominant = 2.35 cm, non-dominant = 2.07 cm).

Children’s peak velocity values (see Supplementary Table A-3) were similar for both directions (ipsilateral = 0.776 m/s, contralateral = 65.9 cm/s), while adults exhibited larger peak velocity values for the ipsilateral (194.4 cm/s), compared to the contralateral target (154.7 cm/s). However, peak velocity values to the ipsilateral target were smaller when performed by the dominant arm (dominant = 107.5 cm/s, non-dominant = 109.0 cm/s). In contrast, average peak velocity to the contralateral target were similar irrespective of arm (dominant = 92.0 cm/s, contralateral = 86.3 cm/s).

Although average shoulder excursion values (see Supplementary Table A-4) were similar for both groups, movements were performed by the dominant arm (ipsilateral = 7.233°, contralateral = 50.149°) differ from those of the non-dominant arm (ipsilateral = 8.587°, contralateral = 48.520°).

Children’s elbow excursion values (see Supplementary Table A-5) were larger for the ipsilateral target as compared to the contralateral target (52.692° vs. 16.717°), whereas adults exhibited smaller excursions for the ipsilateral target (39.331° vs. 63.942°). Interestingly, children showed smaller elbow excursions when moving with the dominant arm (dominant = 33.654°, non-dominant = 35.754°), while young adults showed smaller elbow excursions when movements were performed with the non-dominant arm (dominant = 52.427°, non-dominant = 50.847°). Although average elbow excursion values were similar for both arms (mean = 43.17°) movements toward the ipsilateral target were performed with larger excursions (mean = 46.01°) than movements to the contralateral target (mean = 40.33°). This was, however, influenced by the group performing the movement. Specifically, children’s average elbow excursion values were similar for both arms when moving to the contralateral target (dominant = 15.531°, non-dominant = 17.902°), and larger when moving to the ipsilateral target (dominant = 51.778°, non-dominant = 53.607°). In contrast, average elbow excursion values for adults were larger when moving towards the contralateral target (dominant = 63.260°, non-dominant = 64.624°), but smaller when moving towards the ipsilateral target (dominant = 41.593°, non-dominant = 37.069°).

### Interlimb Coordination

**Figure 3:**
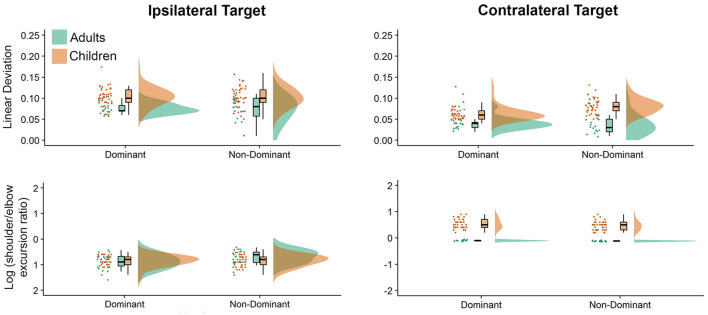
Raincloud plots showing the raw data, data distribution, and five summary statistics for movements to the ipsilateral (left panels) and contralateral directions (right panels) for interjoint coordination variables. Data from preschool children is shown in brown, while data from young adults is plotted in green.

Figure 3 shows interjoint coordination metrics depicted as raincloud plots. The most obvious differences between directions and groups were noted in our interjoint coordination variables. Movements performed to the ipsilateral and contralateral directions showed significant differences, which was displayed in elbow and shoulder joint coordination patterns (i.e., shoulder/elbow excursion ratio, *P* = 0.010), with greater shoulder motion to the contralateral direction. This was consistent with our hand path trajectories measure (Deviation from linearity, *P* < 0.001), which indicated substantially more curved movements for the contralateral target. Hand path curvature was systematically higher for children than adults (*P* < 0.001), which was also reflected by greater Shoulder/Elbow excursion ratios (*P* < 0.001). On average, children exhibited larger deviation from linearity values (see Supplementary Table A-6) for the non-dominant arm (dominant = 0.083, non-dominant = 0.092), while adults exhibited similar deviation from linearity values for adults across arms (dominant = 0.058, non-dominant = 0.055). Adults’ average deviation from linearity was larger for the ipsilateral target (mean = 0.075) than the contralateral target (mean = 0.038), regardless of the arm. In contrast, children’s ipsilateral movements were similar for both the dominant and non-dominant arms (mean = 0.1025), but larger for the non-dominant arm when contralateral movements were performed (ipsilateral = 0.063 vs. contralateral = 0.081).

Last, we examined log (shoulder/elbow excursion ratio) values (see Supplementary Table A-7), which were smaller for the ipsilateral than the contralateral target: adults (−0.755 vs. −0.017), children (−0.845 vs. 0.500). In addition, children exhibited larger log (shoulder/elbow excursion ratio) values for the non-dominant arm (dominant = −0.154, non-dominant = −0.191), whereas adults exhibited larger log (shoulder/elbow excursion ratio) with the dominant arm (dominant = −0.433, non-dominant = −0.339). Also, regardless of arm log (shoulder/elbow excursion ratio) values were smaller when moving to the ipsilateral (dominant = −0.857, non-dominant = −0.742) target than the contralateral target (dominant = 0.270, non-dominant = 0.212).

## Discussion

The main result of the current study is that manual asymmetries in interjoint coordination are apparent for both 6-year-old children and young adults, although young children completed the rapid aiming task by adopting a different strategy than adults. Specifically, adults performed rapid aiming movements to the ipsilateral target using primarily the elbow joint (with a minimal shoulder joint movement) but used both the elbow and shoulder joints for movements to the contralateral target. Children used a similar elbow strategy to adults when performing movements to the ipsilateral target, but used less elbow excursion when performing movements to the contralateral target. Non-dominant arm movements made to the contralateral target were more curved than the dominant arm, resulting from the comparable motion at the shoulder and elbow joints.

### Effects of Target Location

Interestingly, although shoulder excursions were similar across both target locations, there were substantial differences in elbow excursions. The log (shoulder/elbow excursion ratio) presented in Figure 3, clearly demonstrates that both adults and children used a similar coordination strategy when moving to the ipsilateral target (i.e., greater elbow than shoulder excursions). However, when moving to the contralateral target, adults’ shoulder and elbow excursions were similar [as evidenced by log (shoulder/elbow excursion ratio) close to zero], while children used the shoulder joint more than the elbow joint to perform contralateral aiming movements. Thus, to reach the contralateral target, children employed a control strategy that involved more contribution of the shoulder joint when compared to healthy young adults, which is reflective of the level of neuromotor development of the children in question. We postulate that children use more of the proximal joint (i.e., shoulder) to perform aiming movements, as the control of the proximal upper limb (i.e., shoulder) is said to develop before that of the distal upper limb (i.e., hand and fingers; Konczak et al., 1995). We hypothesize that as children gain more experience performing a range of activities of daily living, they learn how to control the relative timing of proximal joint forces, which will then result in the production of stable (i.e., less variable) trajectories and end-points. The result of this experience is that reaching movements to contralateral targets will feature similar amounts of shoulder and elbow excursions.

Based upon the available evidence, it would seem that the control strategies employed by strongly right-handed 6-year-old children differ from that of healthy young adults, in large part because some features of upper limb control are not fully developed at this age. Overall, children’s rapid aiming performance was slower and less coordinated than that of healthy young adults. Indeed, the results of the present study indicate that the only variables that were similar between 6-year-old children and adults were final position error and shoulder excursion. Specifically, it appears that 6-year-old children are unable to exploit non-muscular intersegmental torques required to perform this rapid aiming task, indicating that the control of multiple body axes is still not fully matured at this stage of development.

Empirical research from developing populations also lends support to our hypothesis that the development of reaching metrics is not linear. Overall, there is evidence that the development of a mature-like stereotype trajectory of the hand is accompanied by concomitant changes in angular kinematics (Thelen, 1989; Berthier et al., 1999, 2005; Schneiberg et al., 2002). For example, Schneiberg et al. (2002) studied the movement kinematics and interjoint coordination of 4 and 11-year-old children when reaching for targets at three different distances, with results indicating that the temporal coupling of the shoulder and elbow did not reach levels typically observed in adult populations, regardless of age. According to Schneiberg et al. (2002), not all aspects of reaching develop at the same time, instead, each aspect has its time rate, so that interjoint coordination does not necessarily develop with hand path or trajectory smoothness.

We postulate that the control strategies employed by children in this study arise from two, not mutually exclusive, possibilities. First, from a biomechanical perspective, the multijoint movements employed in the present experiment require the regulation of passive interactive torques, but that the required interaction torques differed depending on whether movements were performed to the contralateral or ipsilateral target. As such, probably, the 6-year-old children are still exploring how best to proficiently control the intersegmental dynamics required to perform the task. Alternatively, the interhemispheric transmission of information relevant to the planning and execution of rapid movements may influence the control strategies employed by 6-year-old children. According to these theories (see Hodges et al., 1997), it is hypothesized that the visual position of the ipsilateral target is processed by the cerebral hemisphere that innervates the musculature of reaching arm. Movements made to ipsilateral space are processed within the same cerebral hemisphere, and thus at least some aspects of the movement can be organized more efficiently and quickly. In contrast, when movements are made to the contralateral target, the afferent and efferent processes are processed in two cerebral hemispheres, which leads to a degradation of task-relevant information (Jones and Elliott, 1988). Given that these latter studies utilized experimental paradigms in which participants focus on a central target, there is cause to question whether the interhemispheric transmission of information theory would hold in the current experiment where participant’s eye movements were not restricted. Future research is required to tease out which of these possibilities account for our results and would benefit from the use of electroencephalography (EEG) to estimate interhemispheric transfer time (Chaumillon et al., 2018) during movements to ipsilateral and contralateral targets.

### Effects of Arm

Consistent with prior literature (van Mier, 2006), movements were faster and more accurate when performed with the dominant right arm. However, these main effects were superseded by the significant interactions between arm and direction, as well as the three-way interactions between the group, arm, and direction. Focusing specifically on manual asymmetries in the 6-year-old children, it was observed that children exhibited more curved trajectories when performing movements to the contralateral target with the non-dominant left arm, compared to the other tested conditions. This is likely an effect of the employed control strategies, such that children’s shoulder excursions were smaller, and elbow excursions were larger when moving to the contralateral target with the non-dominant arm. This combination of effects together led to a more substantial influence on the ratio between shoulder and elbow excursions, which differed from the ratio exhibited by the adult participants. This arrangement of shoulder and elbow coordination (i.e., less shoulder joint movement, but more elbow joint movement) directly limits the possible hand trajectories that an individual can perform.

While speculative, we postulate that the decreased performance by the non-dominant arm in contralateral space is driven by hand preference and the degree of experience that the preferred arm obtains during childhood compared to the non-preferred arm. In comparison to adults who use their preferred arm to reach into contralateral space 30% of the time (Leconte and Fagard, 2004), children aged 6–7 years use their preferred arm for reaching objects, regardless of where they are moving in space (Bryden and Roy, 2006). Thus, it is likely that the increased usage of the dominant right arm (i.e., the motor dominance) provides the child with ample opportunities to learn, *via* trial and error processes, the most efficient joint configurations for a given task. In contrast, the decreased use of the non-dominant left arm reduces the developing child’s ability to explore what the most appropriate control strategies are, leading to joint configurations between the shoulder and elbow, that restrict their movements to curved, rather than straight-line trajectories.

### Practical Implications and Limitations

In considering the results of the current study, we argue that the differences in interjoint coordination reflect the stage of development of 6-year-old children, the origin of which derives from maturational (e.g., hand dominance) and environmental factors (e.g., school-based experience). Specifically, children in the current study have just started elementary school, and as such are learning new ways of interacting with the environment and *via* participation in structured and developmentally appropriate physical education classes where fundamental movement skills and physical competencies can be reinforced through professional advice (Olrich, 2002). Additionally, classroom activities that require the use of computer mouse, cutting with scissors, and drawing further contribute to the development of several motor skills, including motor coordination, fine and gross motor skills, and aiming performance (McManus et al., 1988; Rodrigues et al., 2010). The coordination of several muscles or muscle groups in the performance of rapid aiming movements develops gradually throughout the childhood years, due in large part to the extensive psychomotor experience.

While this study provides important information on movement kinematics and interjoint coordination patterns in 6-year-old children, there are several limitations that researchers should consider. First, we focused our analysis on movement kinematics and interjoint coordination and did not measure torque and muscle activity in the current study. In future work, we will certainly employ these more sophisticated techniques (electromyography to strengthen joint torques) and analysis [Functional Data Analysis (FDA) see Gallivan and Chapman, 2014], as these measures may provide useful information about manual asymmetries during reaching (Schneiberg et al., 2002). Second, if we assume that healthy young adult reaching is reflective of optimal performance, then our data indicate that the control of multiple body axes is not yet fully matured at this stage of development. As such, future research should examine how biomechanical and neurological constraints mediate manual asymmetries, and to investigate how these constraints interact with one another during development. Given the rapid change in motor skill proficiency during the early primary school years, the next step in this line of work would be to investigate the long-term development of motor skills using a sequential design (whereby changes over time can be measured and compared with differences between age groups) across a large swath of the developmental spectrum, as well as examine multiple motor behavior components (e.g., reaching and grasping, object transportation) within the same study.

## Data Availability Statement

The raw data supporting the conclusions of this article will be made available by the authors, without undue reservation.

## Ethics Statement

The studies involving human participants were reviewed and approved by Universidade Federal do ABC Institutional Review Board and San Francisco State University Institutional Review Board. Written informed consent to participate in this study was provided by the participants’ legal guardian when appropriated.

## Author Contributions

LB and RB designed the experiment and formulated the experimental questions. LB, RB, and CH collected the data and revised the final version of the manuscript. CH and LB performed data analysis and statistics and wrote the article.

## Conflict of Interest

The authors declare that the research was conducted in the absence of any commercial or financial relationships that could be construed as a potential conflict of interest.
