# Supplementary material for: Movement Kinematics and Interjoint Coordination Are Influenced by Target Location and Arm in 6-Year-Old Children
Source: Front Hum Neurosci. 2020 Sep 16;14:554378. doi: 10.3389/fnhum.2020.554378 (PMC7533587; doi:10.3389/fnhum.2020.554378)
Supplement: Supplementary file 1 [file Data_Sheet_1.pdf]

APPENDIX:

Tables (**A1 – 7**) show ANOVA results for movement time (A-1), final position error (A-2), peak hand velocity (A-3), deviation from linearity (A-4), shoulder excursion (A-5), elbow excursion (A-6), and Log (shoulder/elbow excursion ratio) (A-7).

Means for Arm (D = dominant, ND = non-dominant), Direction (IL = ipsilateral, CL = contralateral), and Group (C = children, A = adults) are shown in brackets.

Table A-1. ANOVA: Movement time (ms)

**Factor**	**DOF**	***F* value**	***P* value**	η^2_p
Arm (D = 453; ND = 474)	1	7.004	0.010	0.106
Direction (IL = 415; CL = 511)	1	157.796	< .001	0.728
Group (C = 597; A = 329)	1	363.503	< .001	0.860
Direction *x* Group	1	8.460	0.005	0.125
Arm *x* Direction	1	9.225	0.004	0.135

Table A-2. ANOVA: Final position error (cm)

**Factor**	**DOF**	***F* value**	***P* value**	η^2_p
Arm (D = 2.010; ND = 2.272)	1	6.148	0.016	0.094
Arm *x* Direction *x* Group	1	9.378	0.003	0.137

Table A-3. ANOVA: Peak hand velocity (cm/s)

Factor	DOF	*F* value	*P* value	η^2_p
Direction (IL = 13.60; CL = 11.03)	1	274.182	< .001	0.823
Group (C = 7.17; A = 17.45)	1	157.796	< .001	0.728
Direction *x* Group	1	81.010	< .001	0.579
Arm *x* Direction	1	17.030	< .001	0.224

Table A-4. ANOVA: Deviation from linearity

Factor	DOF	*F* value	*P* value	η^2_p
Direction (IL = 0.089; CL = 0.055)	1	118.525	< .001	0.668
Group (C = 0.087; A = 0.057)	1	80.723	< .001	0.578
Arm *x* Group	1	4.611	0.036	0.072
Arm *x* Direction *x* Group	1	5.521	0.022	0.086

Table A-5. ANOVA: Shoulder excursion (º)

Factor	DOF	*F* value	*P* value	η^2_p
Direction (IL = 7.910; CL = 49.335)	1	1660.784	< .001	0.966
Arm *x* Direction	1	8.737	0.004	0.129

Table A-6. ANOVA: Elbow excursion (º)

Factor	DOF	*F* value	*P* value	η^2_p
Group (C = 34.704; A = 51.637)	1	215.406	< .001	0.785
Direction (IL = 46.012; CL = 40.329)	1	94.513	< .001	0.616
Direction *x* Group	1	2686.184	< .001	0.979
Arm *x* Group	1	10.766	0.002	0.154
Arm *x* Direction	1	15.788	< .001	0.211
Arm *x* Direction *x* Group	1	10.912	0.002	0.156

Table A-7. ANOVA: Log (Shoulder/Elbow excursion ratio)

Factor	DOF	*F* value	*P* value	η^2_p
Direction (IL = -0.800; CL = 0.241)	1	50.228	0.010	0.911
Group (C = -0.172; A = -0.386)	1	47.123	< .001	0.457
Direction *x* Group	1	48.501	< .001	0.464
Arm *x* Group	1	13.740	< .001	0.197
Arm *x* Direction	1	14.373	< .001	0.204
